# The Use of Gene Ontology Term and KEGG Pathway Enrichment for Analysis of Drug Half-Life

**DOI:** 10.1371/journal.pone.0165496

**Published:** 2016-10-25

**Authors:** Yu-Hang Zhang, Chen Chu, Shaopeng Wang, Lei Chen, Jing Lu, XiangYin Kong, Tao Huang, HaiPeng Li, Yu-Dong Cai

**Affiliations:** 1 School of Life Sciences, Shanghai University, Shanghai 200444, People’s Republic of China; 2 Institute of Health Sciences, Shanghai Institutes for Biological Sciences, Chinese Academy of Sciences, Shanghai 200031, People’s Republic of China; 3 Institute of Biochemistry and Cell Biology, Shanghai Institutes for Biological Sciences, Chinese Academy of Sciences, Shanghai 200031, People’s Republic of China; 4 College of Information Engineering, Shanghai Maritime University, Shanghai 201306, People’s Republic of China; 5 School of Pharmacy, Key Laboratory of Molecular Pharmacology and Drug Evaluation (Yantai University), Ministry of Education, Collaborative Innovation Center of Advanced Drug Delivery System and Biotech Drugs in Universities of Shandong, Yantai University, Yantai 264005, People’s Republic of China; 6 CAS Key Laboratory of Computational Biology, CAS-MPG Partner Institute for Computational Biology, Shanghai Institutes for Biological Sciences, Chinese Academy of Sciences, Shanghai 200031, People’s Republic of China; Tianjin University, CHINA

## Abstract

A drug’s biological half-life is defined as the time required for the human body to metabolize or eliminate 50% of the initial drug dosage. Correctly measuring the half-life of a given drug is helpful for the safe and accurate usage of the drug. In this study, we investigated which gene ontology (GO) terms and biological pathways were highly related to the determination of drug half-life. The investigated drugs, with known half-lives, were analyzed based on their enrichment scores for associated GO terms and KEGG pathways. These scores indicate which GO terms or KEGG pathways the drug targets. The feature selection method, minimum redundancy maximum relevance, was used to analyze these GO terms and KEGG pathways and to identify important GO terms and pathways, such as sodium-independent organic anion transmembrane transporter activity (GO:0015347), monoamine transmembrane transporter activity (GO:0008504), negative regulation of synaptic transmission (GO:0050805), neuroactive ligand-receptor interaction (hsa04080), serotonergic synapse (hsa04726), and linoleic acid metabolism (hsa00591), among others. This analysis confirmed our results and may show evidence for a new method in studying drug half-lives and building effective computational methods for the prediction of drug half-lives.

## Introduction

A drug is any substance that contributes to the relief of various pathological symptoms, which usually induces a pharmacological change in the human body [1–3]. In pharmacology, a pharmaceutical drug or medicine is defined as the functional component that is extracted from biological material or synthesized by the modern pharmaceutical synthesis industry [4]. Drugs, such as antibiotics, have been regarded as the most effective weapons for preventing various diseases in humans and maintaining health. Once drugs are consumed, they are gradually eliminated or metabolized by a specific hepatic microsomal enzyme system [5–7]. To measure the precise amount of time that a drug is effective and control the proper drug dosage, a specific drug parameter, the drug half-life, serves to standardize the use of drugs and avoid side effects [8, 9].

In pharmacology, the drug biological half-life (usually abbreviated half-life) has been defined as the time required for the human body to metabolize or eliminate 50% of the initial value of the functional drug dosage [10]. Similarly, the plasma half-life, another relevant parameter, is defined as the time that it takes for the concentration of the drug in the blood to decrease by 50% [11]. Generally, the two parameters are not equal, but they are closely related [12]. Considering that the real half-life of a specific drug is difficult to detect and measure in most situations (except for drugs with a high tissue residual ratio such as Digitoxin), we take the plasma half-life of drugs as the reference value [13, 14].

Generally, the half-life of a specific drug is affected by six main factors, including plasma protein binding, pharmacokinetic patterns, renal/hepatic diseases, active metabolites, enterohepatic circulation and the specific distribution of the drug volume [15]. All six factors contribute to the regulation of the biotransformation and excretion of a drug, which are two core mechanisms that affect the half-life of a drug [16].

The plasma protein binding affinity has been reported to contribute to the overall metabolic flow in a drug’s transportation, function and elimination. The binding affinity extensively affects the plasma half-life of drugs, which is easily detected, reinforcing the importance of this factor in practical applications [17, 18]. For example, the drug warfarin is an anticoagulant that has a long half-life because of its high binding affinity for plasma proteins [19]. The pharmacokinetic pattern, another factor, has two main metabolic trends for common drugs, first order kinetics and zero order kinetics. According to first order kinetics, a fixed fraction of a drug will be eliminated in a given unit of time, while for zero order kinetics, a fixed amount of a drug will be excreted. The metabolic variation of the two kinetic patterns are mainly influenced by the different metabolic routes of a drug and the dosage [20]. Because of the limited metabolic ability of human bodies, most drugs follow the first pharmacological pattern of metabolism but follow the zero order kinetic pattern of metabolism for toxic doses [21].

As mentioned above, the metabolic ability of human bodies may alter the pattern and speed of specific drugs [5–7]. In humans, the liver tissue contains various hepatic microsomal enzymes and has been found to be the primary location of drug metabolism [22]. Therefore, the metabolic abilities of the hepatic microsomal enzymes may greatly affect the speed of drug metabolism and may further influence their half-lives [23]. For the elimination processes, the kidney is the junction of the urinary system and the circulating system and also affects the half-life of drugs [24, 25]. For example, the accumulation of aminoglycosides has been confirmed during diseases of the kidney [26]. Considering the functions of the liver and kidney during drug metabolism and elimination, the half-life of a drug may be greatly altered by renal or hepatic pathological conditions.

Apart from the factors above, not all drugs are in their activated states when they are absorbed by the human body. Some drugs need to be changed into an activated state (reactive form) to produce pharmacological effects [27]. For example, the half-life of aspirin is fifteen minutes, while the effective metabolic product of aspirin, salicylic acid, has a half-life as long as two hours. This illustrates the crucial role of a drug’s state during its metabolism and elimination [28, 29]. During enterohepatic circulation, such factors extend the in vivo metabolic route of drugs and may further prolong the half-life of certain drugs [30], while the volume of distribution (the ratio of the plasma concentration to the total quantity of a drug (L/kg)) reflects the overall ability to eliminate certain drugs [31]. In total, all six factors are crucial for the biotransformation and excretion of drugs, reflecting the complex regulatory mechanisms that affect their half-lives.

Based on existing experimental methods, it is difficult and time-consuming to screen and verify the proteins and biological processes that may affect the half-life of a specific drug. Most efforts made toward predicting the half-lives of drugs have been based on drug structures. Turner *et al*. predicted human half-lives for 20 cephalosporins based on constitutional, topological, and quantum-chemicals descriptors [32]. Arnot *et al*. developed two half-life prediction models in human based on molecular fragments and an automated iterative fragment selection method [33]. Lu *et al*. predicted elimination half-life in human by seven machine learning methods and molecular descriptors [34]. However, there are few studies investigating the biological mechanisms that may affect the half-lives of drugs.

Here, we applied a computational method to extract functional gene ontology (GO) terms and biological pathways (KEGG pathways) that may affect the half-life of a specific drug. The enrichment of GO terms and KEGG pathways was used to determine their associations with drugs with known half-life values. A popular feature selection method, minimum redundancy maximum relevance (mRMR), was employed to analyze these features and indicated a role for several important GO terms and KEGG pathways in drug metabolism. An analysis of recent publications confirmed the relevance of some of the GO terms and biological pathways that were predicted to affect drug half-life.

## Materials and Methods

### Materials

The terminal half-life data of 670 drugs collected by Obach *et al*. [35] were used in this study and are provided in S1 Table. According to their half-lives, these drugs were classified into the following five categories: (1) compounds with half-lives less than 1 h; (2) compounds with half-lives between 1 and 4 h; (3) compounds with half-lives between 4 and 12 h; (4) compounds with half-lives between 12 and 24 h; and (5) compounds with half-lives greater than 24 h. After mapping 670 drugs to their PubChem IDs and excluding those without PubChem IDs, we obtained 669 drugs. Because each drug in this study was represented by the enrichment scores of GO terms and KEGG pathways, those without these scores were discarded, resulting in 565 drugs (comprising the set *S*). The distribution of these 565 drugs in the aforementioned five categories is listed in Table 1.

**Table 1:** The distribution of drugs in five half-life categories.

Category label	Half-life (t_1/2_)	Number of drugs
1	Compounds with half-lives less than 1 h	56
2	Compounds with half-lives between 1 and 4 h	231
3	Compounds with half-lives between 4 and 12 h	154
4	Compounds with half-lives between 12 and 24 h	61
5	Compounds with half-lives greater than 24 h	63

### Protein-chemical interactions

In this study, we investigated which GO terms and KEGG pathways were associated with effects on drug half-life. However, it was difficult to quantitatively evaluate the correlation between drugs and GO terms or KEGG pathways, which complicated further analyses. The annotated proteins for each GO term and KEGG pathway were easily obtained from public databases. Once proteins related to a specific drug were identified, the correlation between that drug and a GO term or KEGG pathway was measured using the proteins annotating to the GO term or KEGG pathway and those related to the drug.

**Fig 1:**
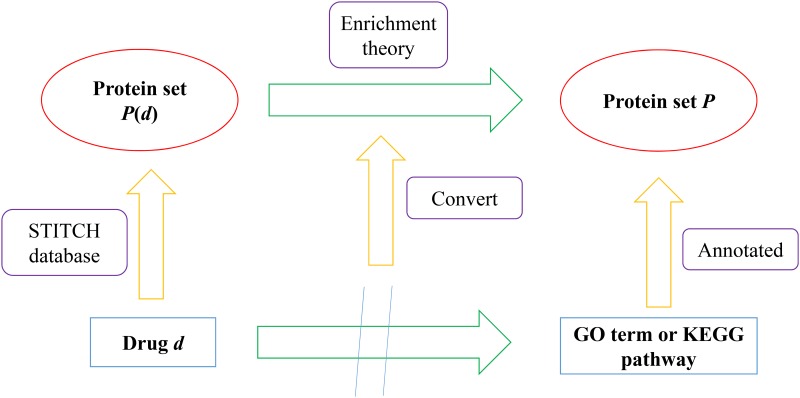
A figure illustrating how the associations between drugs and GO terms or KEGG pathways were measured.

To obtain the proteins related to a specific drug, we downloaded the protein-chemical interactions from the STITCH (Search Tool for Interactions of Chemicals) database [36]. The interactions, including chemical-chemical and protein-chemical interactions, reported in STITCH are derived from experiments, databases and the literature. Thus, they can be used to determine the associations between chemicals and proteins and have been used to address several biological problems [37–43]. We extracted the protein-chemical interactions from the downloaded file ‘protein_chemical.links.v4.0.tsv.gz‘, such that chemicals were members in *S* and proteins were in human tissues, from which a protein set denoted by *P*(*d*) could be accessed for each drug *d* in *S*. Subsequently, the associations between one drug *d* and one GO term or KEGG pathway could be converted to the correlation between two protein sets, where one was *P*(*d*) and the other consisted of proteins annotated with the GO term or KEGG pathway. This idea is illustrated in Fig 1.

### Encoding scheme

As mentioned in Section “Protein-chemical interactions”, based on the protein-chemical interactions and the GO term or KEGG pathway protein annotations, we evaluated the associations between one drug *d* and one GO term or KEGG pathway by measuring the correlation between *P*(*d*) and the set consisting of proteins associated with a GO term or KEGG pathway. Here, we adopted the enrichment theory to quantify the correlation between two protein sets.

#### GO enrichment score

For a given drug *d* and one GO term GO_*j*_, let *P*_*GO*_ denote the set consisting of proteins annotated with GO_*j*_. The GO enrichment score between *d* and GO_*j*_ is defined as the hypergeometric test *P* value [44–46] of *P*(*d*) and *P*_*GO*_, which can be computed by:

$$S_{\text{GO}}\text{(}p\text{,}GO_{j}\text{)}=-{\text{log}}_{10}(\sum_{k=m}^{n}\frac{\left(\begin{array}{c} M\\ k \end{array}\right)\;\left(\begin{array}{c} N-M\\ n-k \end{array}\right)}{\left(\begin{array}{c} N\\ n \end{array}\right)})$$ (1)

where *N* and *M* denote the total number of human proteins and the number of proteins in *P*_*GO*_; *n* and *m* represent the number of proteins in *P*(*d*) and the number of proteins both in *P*(*d*) and *P*_*GO*_. The higher the score is, the stronger the correlation between drug *d* and GO term GO_*j*_. In total, 17,094 GO enrichment scores were calculated in this study for each drug.

#### KEGG enrichment score

A similar method was used to define the KEGG enrichment score, which can measure the associations between drugs and KEGG pathways. Let *P*_*KEGG*_ denote the set consisting of proteins associated with a KEGG pathway *K*_*j*_. The KEGG enrichment score between *d* and *K*_*j*_ is defined to be the hypergeometric test *P* value [46] of *P*(*d*) and *P*_*KEGG*_. Its computational formula is listed below:

$$S_{\text{KEGG}}\text{(}p\text{,}K_{j}\text{)}=-{\text{log}}_{10}(\sum_{k=m}^{n}\frac{\left(\begin{array}{c} M\\ k \end{array}\right)\;\left(\begin{array}{c} N-M\\ n-k \end{array}\right)}{\left(\begin{array}{c} N\\ n \end{array}\right)})$$ (2)

where the parameters *N* and *n* have the same definitions as those in Eq 1, while *M* and *m* denote the number of proteins in *P*_*KEGG*_ and the number of proteins both in *P*(*d*) and *P*_*KEGG*_. Similarly, a large KEGG enrichment score means a strong association between the drug and the pathway. In total, 279 KEGG pathway enrichment scores were calculated in this study for each drug.

The number of GO enrichment scores was much larger than that of the KEGG enrichment scores. Furthermore, the principles for selecting important GO terms and KEGG pathways were not the same. Thus, for each drug in dataset *S*, we obtained separate GO and KEGG enrichment scores resulting in the two datasets of *S*_GO_ and *S*_KEGG_. Drugs in *S*_GO_ were represented by 17,094 GO enrichment scores, and those in *S*_KEGG_ were represented by 279 KEGG enrichment scores.

#### mRMR method

As described in Section “Encoding scheme”, each of the drugs in dataset *S* had 17,094 GO enrichment scores and 279 KEGG enrichment scores that denoted the strength of their association with a given GO term or KEGG pathway. It is obvious that not all GO terms or KEGG pathways have an equal effect on drug half-life; some of them are more important than others. To extract important GO terms and KEGG pathways, the mRMR selection method [47] was employed. This method is useful for analyzing various features and identifying the most important ones, and it has been widely used by investigators to address several biological problems [48–56]. Two excellent criteria were introduced in the mRMR method: Max-Relevance and Min-Redundancy, in which the former criterion guarantees that features with high relevance with targets can receive high ranks, and the latter one guarantees that a feature with lowest redundancies to already-selected features has priority to be selected. Two feature lists can be obtained by the mRMR method, one is called MaxRel feature list and the other is called mRMR feature list. The former list only uses the criterion of Max-Relevance, i.e., features in this list are ranked according to their relevance with targets, while the latter list uses both two criteria to rank features. It is clear that the mRMR feature list can be used to extract an optimal subspace of features for classification, while the MaxRel feature list can be adopted to access important features. Because the purpose of this study is to investigate important factors for determination of drug half-life, we only used the MaxRel feature list yielded by mRMR method. To measure the relevance between a feature *f* and targets, let *x* denote the target variable representing the drugs’ class labels, and *y* denote a variable representing all values under the feature *f*. The relevance between the target and the feature *f* is defined as the mutual information (MI) between *x* and *y*, which can be computed by:

$$I(x,y)=\iint p(x,y)\text{log}\frac{p(x,y)}{p(x)p(y)}dxdy$$ (3)

where *p*(*x*) and *p*(*y*) are the marginal probabilities of *x* and *y* and *p*(*x*,*y*) is the joint probabilistic distribution of *x* and *y*. Accordingly, each feature (one GO term or KEGG pathway) was assigned an MI value, and all features were sorted by the descending order of their MI values in the MaxRel feature list. We selected the GO terms and KEGG pathways with high MI values for further analyses. The mRMR program was downloaded from http://research.janelia.org/peng/proj/mRMR.

## Results and Discussion

### Results of the mRMR method

As described in Section “mRMR method”, a popular feature selection method, the mRMR method, was adopted to extract important GO terms and KEGG pathways that may affect drug half-life. The mRMR program was used to produce MaxRel feature lists for *S*_GO_ and *S*_KEGG_, which are provided in S2 and S3 Tables, respectively. Because our computational power was limited, we only output the first 500 features in the MaxRel feature list for GO terms.

Because GO terms or KEGG pathways with high MI values were more likely to affect drug half-life, we selected a threshold of 0.03 for the MI values of GO terms and 0.013 for the MI values of KEGG pathways. Subsequently, we obtained 23 GO terms and 18 KEGG pathways, which are listed in Tables 2 and 3, respectively. The biological characteristics and properties are analyzed extensively in the following section, producing several useful and important conclusions or suggestions for the study of drug half-lives.

**Table 2:** Important GO terms obtained by the mRMR method and which may be associated with different drug half-lives.

Order	GO term ID	Name	MI value
1	GO:0015347	sodium-independent organic anion transmembrane transporter activity	0.037
2	GO:0060033	anatomical structure regression	0.036
3	GO:0050998	nitric-oxide synthase binding	0.036
4	GO:0035115	embryonic forelimb morphogenesis	0.035
5	GO:0046972	histone acetyltransferase activity (H4-K16 specific)	0.034
6	GO:0043995	histone acetyltransferase activity (H4-K5 specific)	0.034
7	GO:0043996	histone acetyltransferase activity (H4-K8 specific)	0.034
8	GO:0050805	negative regulation of synaptic transmission	0.034
9	GO:0042364	water-soluble vitamin biosynthetic process	0.032
10	GO:0001533	cornified envelope	0.031
11	GO:0008504	monoamine transmembrane transporter activity	0.031
12	GO:0021853	cerebral cortex GABAergic interneuron migration	0.031
13	GO:0021830	interneuron migration from the subpallium to the cortex	0.031
14	GO:0021894	cerebral cortex GABAergic interneuron development	0.031
15	GO:0021534	cell proliferation in hindbrain	0.031
16	GO:0001965	G-protein alpha-subunit binding	0.03
17	GO:1901386	negative regulation of voltage-gated calcium channel activity	0.03
18	GO:0021924	cell proliferation in external granule layer	0.03
19	GO:0021930	cerebellar granule cell precursor proliferation	0.03
20	GO:0046341	CDP-diacylglycerol metabolic process	0.03
21	GO:0019992	diacylglycerol binding	0.03
22	GO:0003881	CDP-diacylglycerol-inositol 3-phosphatidyltransferase activity	0.03
23	GO:0090177	establishment of planar polarity involved in neural tube closure	0.03

**Table 3:** Important KEGG pathways obtained by the mRMR method and which may associated with different drug half-lives.

Order	KEGG pathway ID	Name	MI value
1	hsa04080	Neuroactive ligand-receptor interaction	0.026
2	hsa00400	Phenylalanine, tyrosine and tryptophan biosynthesis	0.024
3	hsa05322	Systemic lupus erythematosus	0.02
4	hsa04726	Serotonergic synapse	0.018
5	hsa00591	Linoleic acid metabolism	0.017
6	hsa05213	Endometrial cancer	0.016
7	hsa00531	Glycosaminoglycan degradation	0.016
8	hsa04146	Peroxisome	0.016
9	hsa00100	Steroid biosynthesis	0.015
10	hsa00603	Glycosphingolipid biosynthesis—globo serie	0.015
11	hsa04530	Tight junction	0.014
12	hsa04666	Fc gamma R-mediated phagocytosis	0.014
13	hsa00130	Ubiquinone and other terpenoid-quinone biosynthesis	0.013
14	hsa04610	Complement and coagulation cascades	0.013
15	hsa00240	Pyrimidine metabolism	0.013
16	hsa04020	Calcium signaling pathway	0.013
17	hsa04725	Cholinergic synapse	0.013
18	hsa00280	Valine, leucine and isoleucine degradation	0.013

### Analysis of important GO terms and KEGG terms for drug half-life

**Fig 2:**
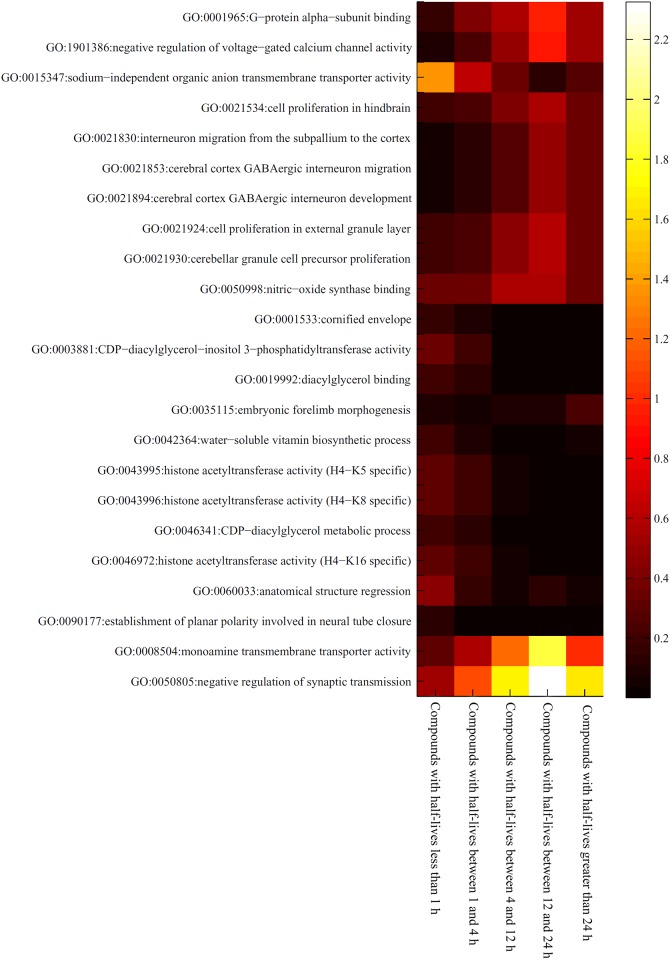
Heat map of the level values for important GO terms in five drug half-life categories. The rows represent GO terms and the columns represent the drug half-life categories.

**Fig 3:**
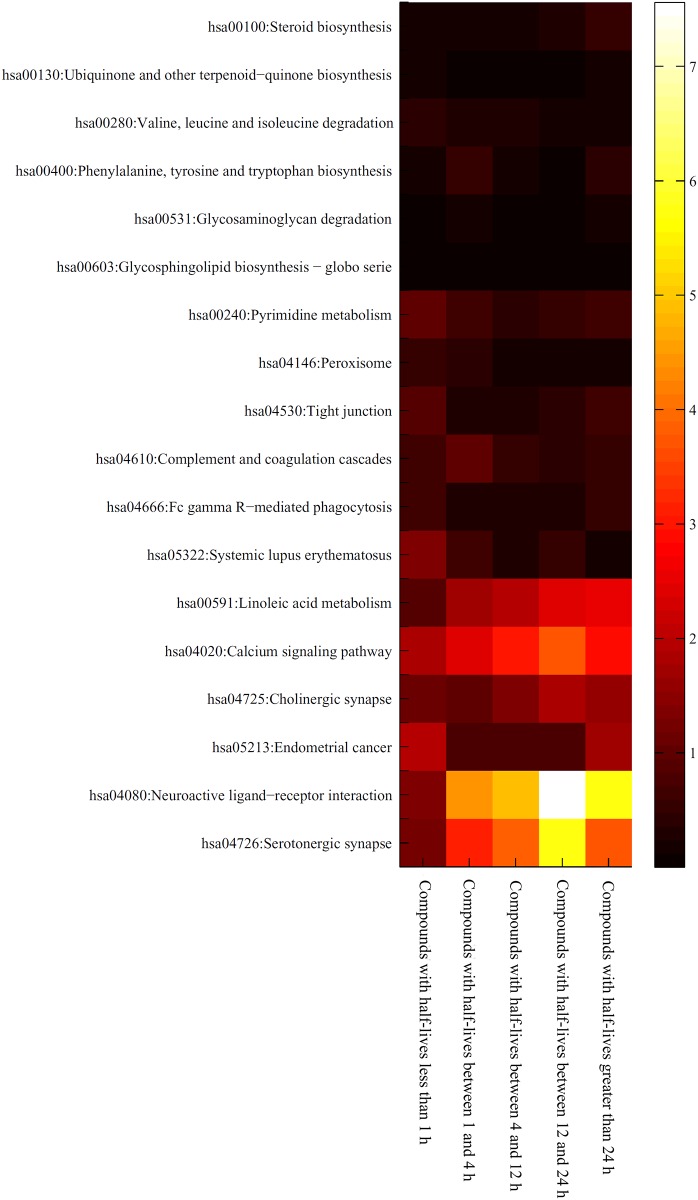
Heat map of the level values for important KEGG pathways in five categories of drug half-life categories. The rows represent KEGG pathways and the columns represent drug half-life categories.

As mentioned in Section “Results of the mRMR method”, 23 GO terms and 18 KEGG pathways were identified that may have an effect on the half-lives of drugs. However, it is difficult to analyze these GO terms and KEGG pathways because drugs with different half-lives received different enrichment scores, even those drugs belonging to the same half-life category. To obtain a better understanding of the associations between a GO term or KEGG pathway and a half-life category, we calculated a “level value” for each GO term or KEGG pathway and each category. The level value was the mean of the enrichment scores for drugs in the category under the GO term or KEGG pathway. The level values for GO terms and KEGG pathways are provided in S4 and S5 Tables, respectively. In addition, for easier visualization, we plotted two heat maps of these values, one is for GO terms (shown in Fig 2), and the other is for KEGG pathways (shown in Fig 3). It can be observed that some GO terms and KEGG pathways are strongly associated with certain half-life categories. Table 4 lists the related GO terms and KEGG pathways for each half-life category. These are discussed in detail in the following sections.

**Table 4:** The relationship between different drug half-lives and GO terms and KEGG pathways discovered in this study.

Half-life (t_1/2_)	Related GO terms	Related KEGG pathways
Compounds with half-lives less than 1 h	GO: 0046972, GO: 0043995, GO:0015347	hsa05322
Compounds with half-lives between 1 and 4 h	---	hsa00400, hsa00531, hsa04610
Compounds with half-lives between 4 and 12 h	GO: 0050998	---
Compounds with half-lives between 12 and 24 h	GO: 0050805, GO: 0008504, GO: 1901386, GO: 0021924	hsa04080, hsa04726
Compounds with half-lives greater than 24 h	GO: 0035115	hsa00591, hsa00100

#### GO terms and KEGG pathways related to compounds with half-lives less than 1 h

Nearly all the GO terms and KEGG pathways contributed to the metabolism and elimination of compounds with half-lives less than 1 h. Considering that both the intake and excretion of drugs require a significant amount of time, drugs that have a half-life less than 1 hour either have a rapid biotransformation process or lack one altogether. GO terms (**GO: 0046972, GO: 0043995**) that contributed to histone trans-acetylation processes were found to participate in the metabolism of drugs with half-lives less than 1 hour. Histone acetylation and deacetylation processes have been reported to be regulated by specific activators and inhibitors [57, 58]. Most of the drugs that were associated with these two GO terms have been reported to have half-lives less than 1 h. For example, experimental data from rats indicated that the antitumor drug TSA has a half-life of approximately 6 minutes and a 50 μM dosage is completely inactivated within 40 minutes, thus validating our classification and prediction [59]. Additionally, some of the drugs with half-lives less than 1 hour are enriched for sodium−independent organic anion transmembrane transporter activity (**GO:0015347**), as shown in Fig 2. According to recent publications, drugs that are associated with this biological process have very short half-lives [60, 61]. Niacin and Alprostadil are two classical drugs that are associated with this process and both of their half-lives are short (20–45 minutes for Niacin and approximately 42 seconds for Alprostadil (PGE1)) [61–63]. KEGG pathways enriched in drugs with half-lives less than one hour included the systemic lupus erythematosus pathway (**hsa05322**). Drugs used to treat systemic lupus erythematosus such as prednisone, also have a very short half-life (plasma half-life of less than 1 hour) [64], which is consistent with our results.

#### GO terms and KEGG pathways related to compounds with half-lives between 1 and 4 h

Unlike drugs with half-lives less than 1 hour, fewer GO terms and KEGG pathways were associated with drugs that have half-lives between 1 and 4 hours. These drugs were enriched in only 3 KEGG pathways (**hsa00400**, **hsa00531** and **hsa04610**) and no GO terms. The KEGG pathway hsa00400 describes phenylalanine, tyrosine and tryptophan biosynthesis. Tetrahydrobiopterin (modified as sapropterin dihydrochloride in drugs) is mainly applied for the treatment of specific diseases such as tetrahydrobiopterin deficiency and neurotransmitter related disorders in the nervous system [65, 66]. The half-life of orally administered sapropterin has been reported to be 4 hours, which is consistent with our prediction [67]. The KEGG pathway hsa00531 describes the glycosaminoglycan degradation biological process. This process is associated with drugs such as chondroitin sulfate, elosulfase alfa and nadroparin [68–70]. Chondroitin sulfate reduces the fat in the blood stream [71, 72]. Considering its chemical nature, a sulfated glycosaminoglycan with a half-life of less than 4 hours, the metabolism and elimination processes of this drug likely involve the predicted KEGG pathway [73, 74]. The KEGG pathway for complement and coagulation cascades (hsa04610) also functions for intercellular substances, which suggests that this metabolic route may be utilized by related drugs. The coagulation factor VIIa is a significant component of the coagulation cascades and definitely participates in the predicted KEGG pathway [75]. This and similar drugs have a half-life of exactly 3.5 hours (between 1 h and 4 h), thereby confirming our prediction of the involvement of this KEGG pathway [76, 77].

#### GO terms and KEGG pathways related to compounds with half-lives between 4 and 12 h

Only one GO term (**GO:0050998**) was enriched for compounds with a half-life between 4–12 hours. GO:0050998 describes nitric−oxide synthase binding activity. Nitric oxide itself is a drug with a half-life of a few seconds in the blood [78]. However, a group of drugs that contribute to the synthesis of nitric-oxide and may participate with the synthase binding associated pathways have been confirmed to have half-lives between 4 and 12 hours [79]. For example, NXN-188 has been confirmed to be associated with the nitric-oxide synthase binding processes and has a corresponding half-life of more than four hours (8–10 hours). Thus, this result also verifies our prediction and classification [80, 81] for this drug. No KEGG pathways were enriched for drugs with half-lives of 4–12 hours.

#### GO terms and KEGG pathways related to compounds with half-lives between 12 and 24 h

Compared to compounds with half-lives between 4 and 12 hours, more GO terms and KEGG pathways were enriched for drugs with half-lives between 12 and 24 hours. As shown in Fig 2, we identified two GO terms (GO: 0050805; GO: 0008504) that may be related to compounds with half-lives between 12 and 24 hours. A negative regulation of synaptic transmission (**GO: 0050805**) has been shown to be regulated by various drugs such as imipramine, alfentanil and anileridine. Imipramine is a common antidepressant drug that has a half-life of 20 hours, which agrees with our prediction. [82, 83]. The GO term **GO: 0008504** refers to monoamine transmembrane transporter activity. The drug transdermal selegiline is involved in this process and has been reported to have a half-life of 18–25 hours, indicating that our prediction was accurate [84, 85]. The term **GO: 1901386** is associated with voltage-gated calcium channels, which are targeted by various drugs [86–88]. Flecainide is a crucial antiarrhythmic drug that regulates the voltage-gated calcium channel and has a specific half-life of 12–27 hours in normal pathological conditions [89–91]. Another GO term, **GO: 0021924**, refers to cell proliferation in the external granule layer, which has been reported to be targeted by the drug methylazoxymethanol. This drug has a half-life of 12 hours in solution [92, 93]. Several enriched KEGG pathways were associated with drugs in the 12–24 hour half-life category. **Hsa04080** describes neuroactive ligand-receptor interactions, which are targeted by drugs such as hydroxyzine [94]. The half-life of hydroxyzine has been reported to be as short as 3 hours; however, the hydroxyzine derivative pamoate has been shown to have a half-life of approximately 20 hours [95, 96]. From the heat maps in Fig 3, it is evident that compounds with half-lives greater than 1 hour (especially compounds with half-lives between 12 and 24 hours) are enriched in this KEGG pathway. The serotonergic synapse pathway (**hsa04726**) is affected by trimipramine, an important antihistamine and sedative with an exact half-life of 23–24 hours [97, 98]. Fig 3 also shows that compounds with half-lives between 12 and 24 hours are enriched in this KEGG pathway, which supports the accuracy and efficacy of our prediction.

#### GO terms and KEGG pathways related to compounds with half-lives greater than 24 h

Some drugs have very long half-lives that are greater than 24 hours. Only one GO term has been predicted to be associated with such long-acting drugs. The term **GO: 0035115** refers to embryonic forelimb morphogenesis. It is well known that the Hedgehog pathway contributes to normal developmental processes in the human embryo. Therefore, Hedgehog pathway inhibitors such as GDC-0449 and AAG are strongly associated with this GO term [99, 100]. Based on recent publications, the half-lives of these drugs are longer than a day (more than 7 days and 5 days for GDC-0449 and AAG, respectively) [100, 101]. Unlike the single enriched GO term, more KEGG pathways were associated with the metabolism of drugs whose half-lives were greater than 24 hours. Linoleic acid metabolism (**hsa00591**) is a crucial pathway for fat metabolism that is used in our daily lives [102]. This process is targeted by two derivatives of linoleic acid, di-homo-gamma-linolenic acid and alpha-linolenic acid. The half-lives of both these linoleic acid derivatives are greater than 24 hours (more than 60 hours for both di-homo-gamma-linolenic acid and alpha-linolenic acid) [103, 104]. Another similar metabolic process, steroid biosynthesis (**hsa00100**), was also enriched for drugs with half-lives greater than 24 hours. Pathways for steroid synthesis have been reported to be associated with rheumatoid arthritis. The drug aurothioglucose, which has been used to treat rheumatoid arthritis, has a half-life of 3–27 days, which is consistent with both our prediction and classification [105, 106].

## Conclusions

This study used the mRMR method to investigate the important GO terms and KEGG pathways that may affect a drug’s half-life. The GO terms and KEGG pathways identified may provide new insights for studying drug half-life and help us build effective prediction models for drug half-lives. We hope that this study can promote pharmacological studies of the drug metabolism mechanism and expand the understanding of half-life-associated biological processes. In future, we will make our efforts in the following two points: (1) Effective models for prediction of drug half-life using some advanced machine learning algorithms [107, 108] can be built based on the extracted GO terms and KEGG pathways; (2) Refined half-life analysis of drugs on certain disease using abundant known information of this disease, such as disease-related target proteins, disease-related microRNA [109, 110], etc.

## Supporting Information

S1 Table 670 drugs and their half-lives. (PDF)

S2 Table The MaxRel feature list with the top 500 GO terms. (PDF)

S3 Table The MaxRel feature list with 278 KEGG pathways. (PDF)

S4 Table Level values of the important GO terms for drugs with different half-lives. (PDF)

S5 Table Level values of the important KEGG pathways for drugs with different half-lives. (PDF)
