# Supplementary material for: The Use of Gene Ontology Term and KEGG Pathway Enrichment for Analysis of Drug Half-Life
Source: PLoS One. 2016 Oct 25;11(10):e0165496. doi: 10.1371/journal.pone.0165496 (PMC5079577; doi:10.1371/journal.pone.0165496)
Supplement: S1 Table — (PDF) [file pone.0165496.s001.pdf]

S1 Table. 670 drugs and their half-lives

Name	PubChem ID	t1/2(h)
Indocyanine_Green	19190	0.065
Fluorouracil,5-	3385	0.12
Dolasetron	60654	0.13
Esmolol	59768	0.15
Artesunate	5464098	0.22
Acetylsalicylic_acid	2244	0.26
Clevidipine	153994	0.3
Irofulven	148189	0.3
Atracurium	47319	0.31
Doxifluridine	18343	0.35
Azacitidine	9444	0.36
Carmustine	2578	0.37
Argatroban	152951	0.4
Isoproterenol	3779	0.41
UNII-QX62KLI41N	175693	0.42
Beclomethasone dipropionate	21700	0.5
Ethacrynic_acid	3278	0.5
Nelarabine	3011155	0.5
Cephalexin	27447	0.57
Iloprost	6443959	0.57

Decitabine	451668	0.58
Omeprazole	4594	0.58
Betamipron	71651	0.59
Quercetin	5280343	0.6
5-aminosalicylic_acid	4075	0.61
Methicillin	6087	0.64
Dichloroacetic_acid	6597	0.65
7-Alpha,19-Methylnortestosterone	52944007	0.65
R-Apomorphine	6005	0.68
Fluvastatin	1548972	0.7
Nafcillin	8982	0.7
Oxacillin	6196	0.7
Penicillin_G	5904	0.7
Dalfopristin	21943991	0.74
Cefamandole	456255	0.75
Cocaine	5760	0.76
Vorinostat	5311	0.76
Flumazenil	3373	0.78
Pravastatin	54687	0.78
Tirapazamine	33776	0.78
Zonampanel	148200	0.78
Allopurinol	2094	0.8

Milrinone	4197	0.8
Remifentanil	60815	0.8
Sch34343	72445	0.8
Cefoxitin	441199	0.81
Mivacurium	5281042	0.83
Phenoxymethylpenicillin	6869	0.84
Cephradine	38103	0.85
Cilastatin	6435415	0.86
Fenoterol	3343	0.87
Quinupristin	23724509	0.87
Repaglinide	65981	0.87
Dicloxacillin	18381	0.88
Clavulanic_Acid	5280980	0.9
Olsalazine	6003770	0.9
Phenethicillin	272833	0.91
Phenacetin	4754	0.92
Cephalothin	6024	0.95
Imipenem	104838	0.95
Panipenem	72015	0.96
Piperacillin	43672	0.96
Nitrofurantoin	5353830	0.97
Abacavir	441300	1

Cefcanel	68666	1
Fenoximone	53708	1
Gemcitabine	60750	1
Hydralazine	3637	1
Indinavir	5362440	1
Isoniazide	3767	1
Lansoprazole	3883	1
Melphalan	460612	1
6-Mercaptopurine	667490	1
Meropenem	441130	1
Rabeprazole	5029	1
SarCNU	100773	1
Ticarcillin	36921	1
Amdinocillin	36273	1.1
Amoxicillin	33613	1.1
Biapenem	71339	1.1
Carbenicillin	20824	1.1
Cefadroxil	47965	1.1
Cefuroxime	5479529	1.1
Chlorambucil	2708	1.1
Cicaprost	5311044	1.1
Levosimendan	3033825	1.1

Mebendazole	4030	1.1
Naloxone	5284596	1.1
Sulbactam	130313	1.1
Tolcapone	4659569	1.1
Zaleplon	5719	1.1
Azlocillin	37625	1.2
Bumetanide	2471	1.2
Cefatrizine	40073	1.2
Cefotaxime	5742673	1.2
Cefprozil	5281006	1.2
Cephapirin	30699	1.2
Dexloxiglumide	65937	1.2
Mezlocillin	656511	1.2
Nefazodone	4449	1.2
Pentoxifylline	4740	1.2
Repinotan	198757	1.2
Sulbenicillin	39031	1.2
Zalcitabine	24066	1.2
Folinic_acid	143	1.3
Iothalamic_acid	3737	1.3
KRN5500	6440176	1.3
Levodopa	6047	1.3

Metrizoate	2528	1.3
Neostigmine	4456	1.3
Nimodipine	4497	1.3
Piretanide	4849	1.3
Propylthiouracil	657298	1.3
Selegiline	26757	1.3
Zidovudine	35370	1.3
Ampicillin	6249	1.4
Diclofenac	3033	1.4
Didanosine	50599	1.4
Flucloxacillin	21319	1.4
Indomethacin	3715	1.4
mevinolinic_acid	64727	1.4
Morphine-6-Glucuronide	5360621	1.4
Rivastigmine	77991	1.4
Stavudine	18283	1.4
UK-240,255	Not_available	1.4
Vecuronium_Bromide	39764	1.4
Aztreonam	5742832	1.5
Cefmetazole	42008	1.5
Ceftizoxime	6533629	1.5
Cephaloridine	5773	1.5

Cocaethylene	65034	1.5
Diprafenone	71249	1.5
Fostriecin	6436285	1.5
Iohexol	3730	1.5
Moclobemide	4235	1.5
Nateglinide	5311309	1.5
Nizatidine	3033637	1.5
Pyridostigmine	4991	1.5
Temozolomide	5394	1.5
Adefovir	60172	1.6
Alfentanil	51263	1.6
Alosetron	2099	1.6
BMS-214662	448545	1.6
Carumonam	6540466	1.6
Fluorescein	16850	1.6
Hydrocortisone	5754	1.6
Ibuprofen	3672	1.6
SureCN6136116	9830010	1.6
Lidocaine	3676	1.6
Melagatran	183797	1.6
Methohexital	9034	1.6
Rocuronium	441290	1.6

Thionylan(Methapyrilene)	4098	1.6
Tirofiban	60947	1.6
Toloxatone	34521	1.6
Cefazolin	33255	1.7
Cefpirome	5479539	1.7
Diprophylline	3182	1.7
Doxacurium_chloride	5284552	1.7
Estradiol	5757	1.7
Gadoversetamide	444013	1.7
Isosorbide_Dinitrate	6883	1.7
Meptazinol	41049	1.7
Metformin	4091	1.7
Sumatriptan	5358	1.7
Tomopenem	9809656	1.7
Zanamivir	60855	1.7
Zolpidem	5732	1.7
Cefoperazone	44185	1.8
Ceftazidime	5481173	1.8
Cerivastatin	446156	1.8
Diatrizoic_acid(Amidotrizoate)	2140	1.8
Edrophonium	3202	1.8
Enprofylline	1676	1.8

Imipramine_N-oxide	65589	1.8
Losartan	3961	1.8
Oseltamivir_acid	449381	1.8
Papaverine	4680	1.8
Pimobendan	4823	1.8
Prilocaine	4906	1.8
Spectinomycin	15541	1.8
Bevantolol	2372	1.9
Cefepime	5479537	1.9
Fosfomycin	446987	1.9
Isosorbide-2-Mononitrate	62989	1.9
Naltrexone	5360515	1.9
1-(4-chlorobenzyl)-3-methylurea	253254	1.9
Nifedipine	4485	1.9
Pancuronium	441289	1.9
Pantoprazole	4679	1.9
Pipercuronium	52944009	1.9
Amrinone	3698	2
Bunazosin	2472	2
Captopril	44093	2
Carboplatin	38904	2
Erythromycin	12560	2

Mepivacaine	4062	2
Morphine	5288826	2
Nicotine	942	2
Prazosin	4893	2
Rosuvastatin	446157	2
Thiopental	3000715	2
Tobramycin	36294	2
Tubocurarine	6000	2
Adinazolam	37632	2.1
Cefetamet	5487888	2.1
Clindamycin	29029	2.1
Eprosartan	5281037	2.1
Ergotamine	8223	2.1
Fosfluconazole	214356	2.1
Iopamidol	65492	2.1
Kanamycin	6032	2.1
Ketoprofen	3825	2.1
Lamifiban	3038484	2.1
Penciclovir	4725	2.1
Propafenone	4932	2.1
Ranitidine	3001055	2.1
Suprofen	5359	2.1

Cimetidine	2756	2.2
Dexmedetomidine	5311068	2.2
Eniporide	6433092	2.2
Etilefrine	3306	2.2
Glyburide	3488	2.2
Moxonidine	4810	2.2
Pindolol	4828	2.2
Rizatriptan	5078	2.2
Ropivacaine	71273	2.2
Timolol	33624	2.2
Bufuralol	71733	2.3
Hydromorphone	5284570	2.3
Ketobemidone	10101	2.3
Methylprednisolone	6741	2.3
Miglitol	441314	2.3
Quinaprilat	107994	2.3
Topotecan	60700	2.3
Tranexamic_acid	5526	2.3
Verlukast	6509849	2.3
Albuterol(Salbutamol)	2083	2.4
Amikacin	37768	2.4
Amsalog	157348	2.4

Carvedilol	2585	2.4
Chlorazepate	2809	2.4
Dibekacin	470999	2.4
Entacapone	5281081	2.4
Estramustine_phosphate	259329	2.4
Methimazole	1349907	2.4
Sisomicin	36119	2.4
Tizanidine	5487	2.4
Tolterodine	443879	2.4
Triamcinolone_Acetonide	6436	2.4
Acetaminophen	1983	2.5
Acyclovir	2022	2.5
Alprenolol	2119	2.5
CB10-277	23606	2.5
Dexrazoxane	71384	2.5
Furosemide	3440	2.5
Methylnaltrexone	5361918	2.5
Olcegepant	6918509	2.5
Pentazocine	441278	2.5
Ribostamycin	46936732	2.5
Tolamolol	37910	2.5
Bambuterol	54766	2.6

Bromfenac	60726	2.6
Cidofovir	60613	2.6
Encainide	48041	2.6
Iopromide	3736	2.6
Ritodrine	33572	2.6
Acarbose	441184	2.7
Thiotepa	5453	2.7
Triazolam	5556	2.7
Alizapride	43008	2.8
Budesonide	5281004	2.8
Clarithromycin	84029	2.8
Famotidine	5702160	2.8
Ketamine	3821	2.8
Verapamil	2520	2.8
Bromopride	2446	2.9
Leuprolide	3911	2.9
Moxalactam_Derivative	17754085	2.9
Prednisone	5865	2.9
Ceforanide	43507	3
Desmopressin	27991	3
Fentanyl	3345	3
Bupivacaine	2474	3.1

Ethambutol	14052	3.1
Midazolam	4192	3.1
Procainamide	4913	3.1
Trimazosin	37264	3.1
Ziprasidone	60854	3.1
Bilobalide	73581	3.2
Buprenorphine	40400	3.2
Cefixime	5362065	3.2
Netilmicin	441306	3.2
Propofol	4943	3.2
Risperidone	5073	3.2
Buflomedil	2467	3.3
Ceftobiprole	6918430	3.3
Dilevalol	134044	3.3
Glipizide	3478	3.3
Isradipine	3784	3.3
Tacrine	1935	3.3
Almotriptan	123606	3.4
Busulphan	2478	3.4
Hydroxyurea	3657	3.4
Ondansetron	4595	3.4
Prednisolone	5755	3.4

Propranolol	4946	3.4
Acebutolol	1978	3.5
DP-b99	9810955	3.5
Etomidate	36339	3.5
Urapidil	5639	3.5
Alcuronium	21917745	3.6
Darifenacin	444031	3.6
Metoprolol	4171	3.6
Zolmitriptan	60857	3.6
Dexamethasone	5743	3.7
Ganciclovir	3454	3.7
Nalbuphine	5311304	3.7
Traxoprodil	219101	3.7
Ciprofloxacin	2764	3.8
Ertapenem	150610	3.8
Ginkgolide_A	115221	3.8
Rifampin	5381226	3.8
Sematilide	58505	3.8
Tezosentan	151174	3.8
Melperone	15387	3.9
Methotrexate	126941	3.9
Rosiglitazone	77999	3.9

Sildenafil	5212	3.9
Codeine	5284371	4
Atropine	174174	4.1
Bosentan	104865	4.1
Doxapram	3156	4.1
Isosorbide-5-Mononitrate	27661	4.1
Nicardipine	4474	4.1
Viloxazine	5666	4.1
Cefodizime	5487888	4.2
Eletriptan	77993	4.2
Eptifibatide	123610	4.2
Flucytosine	3366	4.2
Idazoxan	54459	4.2
Indoramin	33625	4.3
Streptomycin	19649	4.3
Triamterene	5546	4.3
Labetalol	3869	4.4
Nortilidine	162321	4.4
Cefotetan	53025	4.5
Chlormethiazole	10783	4.5
Linezolid	441401	4.5
Scopolamine	5184	4.5

Vardenafil	110634	4.5
Chloramphenicol	5959	4.6
Amsacrine	2179	4.7
m-Chlorophenylpiperazine	1335	4.7
Eltanolone	31402	4.7
Foscarnet	3415	4.7
Gentamicin	3467	4.7
Thalidomide	5426	4.7
Alfuzosin	2092	4.8
Brotizolam	2451	4.8
Butorphanol	6916249	4.8
Methylphenidate	4158	4.8
6-aminohexanoic_acid	564	4.9
Caffeine	2519	4.9
Hexobarbital	3608	4.9
Lormetazepam	13314	4.9
Montelukast	5281040	5
Nefopam	4450	5
Recainam	53084	5
Tilidine	30131	5
Venlafaxine	5656	5
Enoxacin	3229	5.1

Ketorolac	3826	5.1
Metocurine	21233	5.1
Torsemide	41781	5.1
Atomoxetine	54841	5.2
Flavopiridol	5287969	5.2
Ginkgolide_B	65243	5.2
Granisetron	3510	5.2
Hydroflumethazide	3647	5.2
Loxiglumide	60182	5.2
Zopiclone	5735	5.2
Gabapentin	3446	5.3
Galanthamine	9651	5.3
Paricalcitol	5281104	5.3
Clinafloxacin	60063	5.4
Acetylcysteine	12035	5.5
Oxycodone	5284603	5.5
Remoxipride	54477	5.5
Betamethasone	9782	5.6
Diltiazem	39186	5.6
Endralazine	47608	5.6
Lincomycin	656509	5.6
Tropisetron	72165	5.6

Voriconazole	71616	5.6
Dihydroquinidine	91503	5.7
Etoposide	36462	5.7
Benperidol	16363	5.8
Tenofovir	464205	5.8
Tramadol	33741	5.8
Abanoquil	164089	5.9
Hydroxycotinine,3'-	107963	5.9
Methyldopa	38853	5.9
Probenecid	4911	5.9
Tiazofurin	457954	5.9
Finasteride	57363	6
Fluticasone_propionate	444036	6
Atenolol	2249	6.1
Dacarbazine	5353562	6.2
Sulfinpyrazone	5342	6.2
Sotalol	5253	6.3
Acecainide(N-acetylprocainamide)	4342	6.4
Lorcainide	42884	6.5
Nomifensine	4528	6.5
Vancomycin	14969	6.5
Ifosfamide	3690	6.6

Naratriptan	4440	6.6
Quinidine	441074	6.6
Sitafloxacin	73011	6.6
Conivaptan	151171	6.7
Exatecan_mesylate	151114	6.7
3(S)-Hydroxydihydroquinidine	52948881	6.7
Oxazepam	4616	6.7
Telavancin	3081362	6.7
Tamsulosin	129211	6.8
9-Aminocamptothecin	72402	7
Chlortetracycline	24757945	7
Disopyramide	3114	7
Ibutilide	60753	7
Sulfadiazine	5215	7
Tolbutamide	5505	7
Metronidazole	4173	7.1
Primaquine	4908	7.1
Metoclopramide	4168	7.2
Oxybutynin	4634	7.2
Theophylline	2153	7.2
Amphetamine	3007	7.3
Cibenzoline	2747	7.3

Cyclosporine	5284373	7.3
Trazodone	5533	7.3
Sulfisoxazole	5344	7.4
Domperidone	3151	7.5
Oxiracetam	4626	7.5
Clonidine	2803	7.6
Xamoterol	155774	7.7
Lithium_Carbonate	11125	7.8
Cilomilast	151170	7.9
Meperidine	4058	7.9
Promazine	4926	7.9
Cyclophosphamide	2907	8
Piritramide	9331	8
Daptomycin	16129629	8.1
Dofetilide	71329	8.1
Moxifloxacin	152946	8.2
Nitrendipine	4507	8.2
Chlordiazepoxide	2712	8.3
Ethinylestradiol-3-Sulfate	68575	8.4
Pirmenol	4853	8.4
Flupirtine	53276	8.5
Bisaramil	16092094	8.6

Ceftriaxone	5479530	8.6
Fleroxacin	3357	8.6
Levofloxacin	149096	8.8
Nalmefene	5284594	8.8
Rolitetracycline	5282179	8.8
Ofloxacin	4583	8.9
Irinotecan	60838	9
Prochlorperazine	4917	9
Terazosin	5401	9
Lamivudine	60825	9.1
Nadolol	39147	9.2
Diphenhydramine	3100	9.3
Drotaverine	1712095	9.3
Ethinylestradiol-17-Sulfate	67091	9.3
BB83698	9849925	9.4
Fosinoprilat	62956	9.4
Levonorgestrel	13109	9.4
Perphenazine	4748	9.4
Tetracycline	5280962	9.4
Squalamine	72495	9.5
Valsartan	60846	9.5
Trimethoprim	5578	9.6

Ethinyl_estradiol	5991	9.7
Sulfamethoxazole	5329	9.8
Acivicin	294641	9.9
Mexiletine	4178	9.9
Bisoprolol	2405	10
Clozapine	2818	10
Dantrolene	6914273	10
Diflunisal	3059	10
Doxazosin	3157	10
Felodipine	3333	10
Gatifloxacin	5379	10
Gestodene	3033968	10
Glimepiride	3476	10
2-Hydroxyimipramine	108051	10
Nebivolol	71301	10
Oxytetracycline	5280972	10
Reboxetine	65856	10
Tiagabine	60648	10
Chlorpromazine	2726	11
Docetaxel	148124	11
Fludarabine	30751	11
Medroxalol	41835	11

Nisoldipine	4499	11
Paclitaxel	36314	11
Pefloxacin	51081	11
Quinine	8549	11
Romidepsin	5352062	11
Talinolol	68770	11
Tegaserod	5487301	11
Trospectomycin	55886	11
Trovafloxacin	62959	11
Zoledronic_Acid	68740	11
Alprazolam	2118	12
Antipyrine	2206	12
Cetrorelix	25074887	12
Demethylchlortetracycline	54680690	12
Flecainide	3356	12
Ketanserin	3822	12
Methamphetamine	10836	12
Procyclidine	4919	12
Sitagliptin	4369359	12
Telithromycin	3002190	12
Tocainide	38945	12
Valproic_Acid	3121	12

Valspodar	5281884	12
Acetazolamide	1986	13
Aprepitant	151165	13
Mibefradil	60663	13
Paroxetine	43815	13
Saquinavir	60787	13
Tinidazole	5479	13
Dexfenfluramine	3337	14
Doxycycline	5281011	14
Fenspiride	3344	14
Ibandronic_acid(Ibandronate)	60852	14
Irbesartan	3749	14
Meprobamate	4064	14
NK611	6917988	14
Promethazine	4927	14
Sufentanil	41693	14
Doxepin	3158	15
Guanfacine	3519	15
Mirtazapine	4205	15
Terbutaline	5403	15
Amantadine	2130	16
Cladribine	20279	16

Idarubicin	42890	16
Imipramine	3696	16
Micafungin	477468	16
Phencyclidine	6468	16
Teniposide	34698	16
Amitriptyline	2160	17
Azapropazone	26098	17
Betaxolol	2369	17
Cotinine	854019	17
Ixabepilone	23305354	17
Lorazepam	3958	17
Minocycline	54675783	17
Trimetrexate	5583	17
Alphacetylmethadol	22308	18
Anhydrovinblastine	151120	18
Bortezomib	387447	18
Meloxicam	5281106	18
Propoxyphene	10100	18
Temsirolimus	23724530	18
Intoplicine	65954	19
Metolazone	4170	20
Pseudohypericin	5281751	20

larotaxel	6918259	20
Sparfloxacin	60464	20
Telmisartan	65999	20
Bromazepam	2441	21
Men 10755	151896	21
Azelastine	2267	22
Chlorpheniramine	2725	22
Dapsone	2955	22
Desipramine	2995	22
Dexniguldipine	1236	22
Ifetroban	64924	22
Imatinib	5291	22
Pentobarbital	4737	22
Trimipramine	5584	23
Vincristine	5978	23
Biperiden	2381	24
Frovatriptan	77992	24
Isepamicin	3755	24
Flunitrazepam	3380	25
Itraconazole	55283	25
Pentamidine	4735	25
Clomipramine	2801	26

Etoricoxib	123619	26
Nitrazepam	4506	26
Tacrolimus	445647	26
Vinorelbine	60780	26
Caspofungin	2826718	27
Epristeride	68741	27
Perindoprilat	72022	29
Warfarin	54678486	29
Fluconazole	3365	30
Levomepromazine	72287	30
Nortriptyline	4543	30
Desacetylpaclitaxel,4-	6918494	31
Methadone	4095	31
Roquinimex	54676478	31
Doxorubicin	31703	32
KW-2170	5747700	32
7-Methylthiomethylpaclitaxel	6918461	32
Pamidronic_acid(Pamidronate)	4674	32
Citalopram	2771	33
Isoxicam	54677972	33
Tetrahydrocannabinol,D9-	16078	33
Amlodipine	2162	34

Denaverine	71130	34
Gefitinib	123631	34
Haloperidol	3559	35
Vindesine	40839	35
Zotarolimus	23375907	35
Chlorthalidone	2732	36
Epirubicin	41867	36
Maxipost	214350	37
Rifabutin	6323490	37
Clonazepam	2802	38
Digoxin	2724385	38
Enalaprilat	5462501	39
Palonosetron	148211	39
Anidulafungin	166548	40
Diazepam	3016	42
Hypericin	5281051	42
Lisinopril	5362119	42
Trabectedin	108150	44
Letrozole	3902	45
Ribavirin	37542	45
Tesaglitazar	208901	45
Topixantrone	219022	45

Chlorpropamide	2727	46
Desmethyldiazepam	2997	46
Diazoxide	3019	48
Tigecycline	5282044	48
Genaconazole	60741	49
Eritoran	6912404	51
Maprotiline	4011	51
Solifenacin	216457	52
Mitoxantrone	4212	53
Nevirapine	4463	53
Terodiline	23480	56
Colchicine	6167	58
DHAPaclitaxel	6918473	60
Atovaquone	74989	63
Tenoxicam	5312154	67
Vinblastine	241903	67
Azithromycin	447043	69
Aripiprazole	60795	75
Azimilide	9571004	79
Troxacitabine	151173	82
Phenobarbital	4763	99
Quinacrine	237	120

Pyrimethamine	4993	140
61036-64-4	16132315	150
Dalbavancin	23724878	170
Digitoxin	441207	180
Delorazepam	17925	200
Risedronicacid(Risedronate)	5245	200
Chloroquine	2719	570
PNU-145156E	132977	730
7-hydroxystaurosporine	72271	790
Amiodarone	2157	820
Hydroxychloroquine	3652	850
Suramin	5361	1200
