# Supplementary material for: The Use of Gene Ontology Term and KEGG Pathway Enrichment for Analysis of Drug Half-Life
Source: PLoS One. 2016 Oct 25;11(10):e0165496. doi: 10.1371/journal.pone.0165496 (PMC5079577; doi:10.1371/journal.pone.0165496)
Supplement: S2 Table — (PDF) [file pone.0165496.s002.pdf]

S2 Table. The MaxRel feature list with the top 500 GO terms

Order	GO terms	MI value
1	GO:0015347	0.037
2	GO:0060033	0.036
3	GO:0050998	0.036
4	GO:0035115	0.035
5	GO:0046972	0.034
6	GO:0043995	0.034
7	GO:0043996	0.034
8	GO:0050805	0.034
9	GO:0042364	0.032
10	GO:0001533	0.031
11	GO:0008504	0.031
12	GO:0021853	0.031
13	GO:0021830	0.031
14	GO:0021894	0.031
15	GO:0021534	0.031
16	GO:0001965	0.03
17	GO:1901386	0.03
18	GO:0021924	0.03
19	GO:0021930	0.03

20	GO:0046341	0.03
21	GO:0019992	0.03
22	GO:0003881	0.03
23	GO:0090177	0.03
24	GO:0060080	0.029
25	GO:0031528	0.029
26	GO:0019866	0.029
27	GO:0048149	0.029
28	GO:0051586	0.029
29	GO:0051582	0.029
30	GO:0051944	0.029
31	GO:0044425	0.029
32	GO:0043967	0.029
33	GO:0021892	0.029
34	GO:0060073	0.029
35	GO:0005335	0.029
36	GO:0019811	0.029
37	GO:0021941	0.029
38	GO:0021769	0.029
39	GO:0048755	0.029
40	GO:0014848	0.028

41	GO:0014832	0.028
42	GO:0080130	0.028
43	GO:0015301	0.028
44	GO:0097254	0.028
45	GO:0016021	0.028
46	GO:0001159	0.028
47	GO:0097154	0.028
48	GO:0035024	0.028
49	GO:0034776	0.028
50	GO:0055069	0.027
51	GO:0001711	0.027
52	GO:0070573	0.027
53	GO:0072340	0.027
54	GO:0016999	0.027
55	GO:0015222	0.027
56	GO:0042403	0.027
57	GO:0016098	0.027
58	GO:0018675	0.027
59	GO:0018676	0.027
60	GO:0052741	0.027
61	GO:0019113	0.027

62	GO:0060198	0.027
63	GO:0005816	0.027
64	GO:0004728	0.027
65	GO:0048854	0.027
66	GO:0015333	0.027
67	GO:0022897	0.027
68	GO:0051937	0.027
69	GO:0071339	0.027
70	GO:0044665	0.027
71	GO:0001985	0.027
72	GO:0060071	0.027
73	GO:0090175	0.027
74	GO:0035567	0.027
75	GO:0005743	0.026
76	GO:0035136	0.026
77	GO:0043163	0.026
78	GO:0045229	0.026
79	GO:0060484	0.026
80	GO:0044306	0.026
81	GO:0007195	0.026
82	GO:0004866	0.026

83	GO:0061325	0.026
84	GO:0015742	0.026
85	GO:0051058	0.026
86	GO:0046580	0.026
87	GO:0021826	0.026
88	GO:0021843	0.026
89	GO:0035097	0.026
90	GO:0005427	0.026
91	GO:0015322	0.026
92	GO:0035240	0.026
93	GO:0042249	0.026
94	GO:0061135	0.026
95	GO:0030414	0.026
96	GO:0035634	0.026
97	GO:0006103	0.026
98	GO:0042220	0.026
99	GO:0019905	0.026
100	GO:0005119	0.026
101	GO:0016323	0.026
102	GO:0043266	0.025
103	GO:0097195	0.025

104	GO:0051590	0.025
105	GO:0006590	0.025
106	GO:0009236	0.025
107	GO:0004952	0.025
108	GO:0021801	0.025
109	GO:0034750	0.025
110	GO:0030136	0.025
111	GO:0006836	0.025
112	GO:0051588	0.025
113	GO:0001591	0.025
114	GO:0090493	0.025
115	GO:0090494	0.025
116	GO:0051583	0.025
117	GO:0051934	0.025
118	GO:0048148	0.025
119	GO:1900273	0.025
120	GO:0070986	0.025
121	GO:0070546	0.025
122	GO:0003057	0.025
123	GO:0061324	0.025
124	GO:0060492	0.025

125	GO:0060424	0.025
126	GO:1901963	0.025
127	GO:0060423	0.025
128	GO:1900271	0.024
129	GO:0090346	0.024
130	GO:0090350	0.024
131	GO:0090347	0.024
132	GO:0090349	0.024
133	GO:0090348	0.024
134	GO:0090345	0.024
135	GO:0097267	0.024
136	GO:0042542	0.024
137	GO:0033234	0.024
138	GO:0030672	0.024
139	GO:0019551	0.024
140	GO:0006106	0.024
141	GO:0019550	0.024
142	GO:0006532	0.024
143	GO:0004069	0.024
144	GO:0000014	0.024
145	GO:0016805	0.024

146	GO:0043981	0.024
147	GO:0043982	0.024
148	GO:0006397	0.024
149	GO:0005334	0.024
150	GO:0016998	0.024
151	GO:0030424	0.024
152	GO:0005452	0.024
153	GO:0032229	0.024
154	GO:2000353	0.024
155	GO:0014046	0.024
156	GO:0014059	0.024
157	GO:0070016	0.024
158	GO:0030674	0.024
159	GO:0005913	0.024
160	GO:0042713	0.024
161	GO:0051940	0.024
162	GO:0051584	0.024
163	GO:0010996	0.023
164	GO:0015075	0.023
165	GO:0022891	0.023
166	GO:0008038	0.023

167	GO:0005212	0.023
168	GO:0007612	0.023
169	GO:0017075	0.023
170	GO:0021936	0.023
171	GO:0007386	0.023
172	GO:0004937	0.023
173	GO:0032415	0.023
174	GO:0001505	0.023
175	GO:0015844	0.023
176	GO:0000987	0.023
177	GO:0070852	0.023
178	GO:1901160	0.023
179	GO:0007613	0.023
180	GO:0031503	0.023
181	GO:0060004	0.023
182	GO:0019483	0.023
183	GO:0018377	0.023
184	GO:0008214	0.023
185	GO:0006482	0.023
186	GO:0060452	0.023
187	GO:0008449	0.023

188	GO:0032456	0.023
189	GO:0060359	0.023
190	GO:0016818	0.023
191	GO:0016462	0.023
192	GO:0016817	0.023
193	GO:0036022	0.023
194	GO:0036023	0.023
195	GO:0072054	0.023
196	GO:0030997	0.023
197	GO:0072053	0.023
198	GO:0042789	0.023
199	GO:0043679	0.023
200	GO:0030297	0.023
201	GO:0003746	0.023
202	GO:0060708	0.022
203	GO:0042417	0.022
204	GO:0005070	0.022
205	GO:0044340	0.022
206	GO:0035255	0.022
207	GO:0031224	0.022
208	GO:0042939	0.022

209	GO:0034635	0.022
210	GO:0019373	0.022
211	GO:0003025	0.022
212	GO:0021794	0.022
213	GO:0003160	0.022
214	GO:0042428	0.022
215	GO:0001994	0.022
216	GO:0034708	0.022
217	GO:0007212	0.022
218	GO:0030534	0.022
219	GO:0031404	0.022
220	GO:0033238	0.022
221	GO:0035640	0.022
222	GO:0001975	0.022
223	GO:0050982	0.022
224	GO:0008241	0.022
225	GO:0022029	0.022
226	GO:0060158	0.022
227	GO:0060306	0.022
228	GO:0043984	0.022
229	GO:0032784	0.022

230	GO:0061097	0.022
231	GO:0008021	0.022
232	GO:0000781	0.022
233	GO:0070076	0.022
234	GO:0016577	0.022
235	GO:0050966	0.022
236	GO:0097108	0.022
237	GO:0045778	0.022
238	GO:0030594	0.022
239	GO:0050955	0.022
240	GO:0060534	0.022
241	GO:0016721	0.021
242	GO:0004784	0.021
243	GO:0004402	0.021
244	GO:0043014	0.021
245	GO:0071886	0.021
246	GO:0007208	0.021
247	GO:0016907	0.021
248	GO:0046950	0.021
249	GO:0043005	0.021
250	GO:0042094	0.021

251	GO:0008158	0.021
252	GO:0036126	0.021
253	GO:0006214	0.021
254	GO:0046127	0.021
255	GO:0017113	0.021
256	GO:0001978	0.021
257	GO:0031441	0.021
258	GO:0008188	0.021
259	GO:1902589	0.021
260	GO:0009299	0.021
261	GO:0019471	0.021
262	GO:0007197	0.021
263	GO:0047801	0.021
264	GO:0004609	0.021
265	GO:0033169	0.021
266	GO:2000311	0.021
267	GO:0035987	0.021
268	GO:0000149	0.021
269	GO:1901162	0.021
270	GO:0042447	0.021
271	GO:0045987	0.021

272	GO:0009086	0.021
273	GO:0010155	0.021
274	GO:0007188	0.021
275	GO:0048701	0.021
276	GO:0003917	0.021
277	GO:0008127	0.021
278	GO:0009712	0.021
279	GO:0006584	0.021
280	GO:0008559	0.021
281	GO:0080184	0.021
282	GO:0061245	0.021
283	GO:0035088	0.021
284	GO:0007213	0.021
285	GO:0043586	0.021
286	GO:0021997	0.021
287	GO:0016342	0.021
288	GO:0048487	0.021
289	GO:0043605	0.02
290	GO:0050820	0.02
291	GO:0006533	0.02
292	GO:0060120	0.02

293	GO:0009912	0.02
294	GO:0048170	0.02
295	GO:0016338	0.02
296	GO:0004129	0.02
297	GO:0015002	0.02
298	GO:0016676	0.02
299	GO:0016675	0.02
300	GO:0006116	0.02
301	GO:0045076	0.02
302	GO:0010157	0.02
303	GO:0043198	0.02
304	GO:0021800	0.02
305	GO:0021797	0.02
306	GO:0001730	0.02
307	GO:0020027	0.02
308	GO:0061337	0.02
309	GO:0006882	0.02
310	GO:0031280	0.02
311	GO:0007194	0.02
312	GO:0051350	0.02
313	GO:0014075	0.02

314	GO:0033602	0.02
315	GO:0004936	0.02
316	GO:0060916	0.02
317	GO:0055075	0.02
318	GO:0009650	0.02
319	GO:0008290	0.02
320	GO:0003263	0.02
321	GO:0010576	0.02
322	GO:0048551	0.02
323	GO:0008191	0.02
324	GO:0051580	0.02
325	GO:0051865	0.02
326	GO:0050308	0.02
327	GO:0019203	0.02
328	GO:0014820	0.02
329	GO:0060090	0.02
330	GO:0071203	0.02
331	GO:0005887	0.02
332	GO:0021984	0.02
333	GO:0097458	0.02
334	GO:0004673	0.02

335	GO:0061098	0.02
336	GO:0017169	0.02
337	GO:0030010	0.02
338	GO:0014824	0.02
339	GO:0032417	0.02
340	GO:0004521	0.02
341	GO:0006531	0.02
342	GO:0035414	0.02
343	GO:0034235	0.02
344	GO:0048243	0.02
345	GO:0007207	0.02
346	GO:0005815	0.02
347	GO:0021895	0.02
348	GO:0003955	0.02
349	GO:0014072	0.02
350	GO:0043495	0.019
351	GO:0061326	0.019
352	GO:0030194	0.019
353	GO:1900048	0.019
354	GO:0022857	0.019
355	GO:0042373	0.019

356	GO:0004046	0.019
357	GO:0033267	0.019
358	GO:0070602	0.019
359	GO:0070601	0.019
360	GO:0032482	0.019
361	GO:0043175	0.019
362	GO:0097223	0.019
363	GO:0009067	0.019
364	GO:0050974	0.019
365	GO:0015872	0.019
366	GO:0009880	0.019
367	GO:0031226	0.019
368	GO:0032200	0.019
369	GO:0009403	0.019
370	GO:0071615	0.019
371	GO:0018958	0.019
372	GO:0006333	0.019
373	GO:0090179	0.019
374	GO:0090178	0.019
375	GO:0010511	0.019
376	GO:1902302	0.019

377	GO:1902303	0.019
378	GO:0061526	0.019
379	GO:0097150	0.019
380	GO:0051823	0.019
381	GO:1901374	0.019
382	GO:0015870	0.019
383	GO:0031424	0.019
384	GO:0031616	0.019
385	GO:0005915	0.019
386	GO:0072348	0.019
387	GO:0090037	0.019
388	GO:0014061	0.019
389	GO:0006516	0.019
390	GO:0051481	0.019
391	GO:0034111	0.019
392	GO:0030425	0.019
393	GO:0015464	0.019
394	GO:0000783	0.019
395	GO:0000782	0.019
396	GO:0021819	0.019
397	GO:0015697	0.019

398	GO:0005777	0.019
399	GO:0042579	0.019
400	GO:0042053	0.019
401	GO:0042069	0.019
402	GO:0044463	0.019
403	GO:1901533	0.019
404	GO:0001669	0.019
405	GO:0009822	0.019
406	GO:0072672	0.019
407	GO:0021943	0.019
408	GO:0003266	0.019
409	GO:0003264	0.019
410	GO:0090325	0.019
411	GO:0060160	0.019
412	GO:0006538	0.019
413	GO:0001587	0.019
414	GO:0005355	0.019
415	GO:1902017	0.019
416	GO:0001046	0.019
417	GO:0070063	0.019
418	GO:0042427	0.019

419	GO:0004904	0.019
420	GO:0016787	0.019
421	GO:0005640	0.019
422	GO:0071073	0.019
423	GO:0010513	0.019
424	GO:0060482	0.019
425	GO:0060481	0.019
426	GO:0060066	0.019
427	GO:0030432	0.018
428	GO:0006020	0.018
429	GO:0000723	0.018
430	GO:1902652	0.018
431	GO:0044107	0.018
432	GO:0070640	0.018
433	GO:0044108	0.018
434	GO:0036378	0.018
435	GO:0006122	0.018
436	GO:0070603	0.018
437	GO:0048262	0.018
438	GO:0008443	0.018
439	GO:0043649	0.018

440	GO:0007191	0.018
441	GO:0098562	0.018
442	GO:0002005	0.018
443	GO:0060244	0.018
444	GO:1990144	0.018
445	GO:0071279	0.018
446	GO:0005330	0.018
447	GO:1901798	0.018
448	GO:0014807	0.018
449	GO:0004888	0.018
450	GO:0086001	0.018
451	GO:0070471	0.018
452	GO:0003213	0.018
453	GO:0007638	0.018
454	GO:0000302	0.018
455	GO:0072376	0.018
456	GO:0097055	0.018
457	GO:0050890	0.018
458	GO:0007200	0.018
459	GO:0072540	0.018
460	GO:0008395	0.018

461	GO:0008306	0.018
462	GO:0007626	0.018
463	GO:0086091	0.018
464	GO:0007163	0.018
465	GO:0004622	0.018
466	GO:0035229	0.018
467	GO:0035227	0.018
468	GO:0035226	0.018
469	GO:0032886	0.018
470	GO:0016020	0.018
471	GO:0004872	0.018
472	GO:0001986	0.018
473	GO:0002541	0.018
474	GO:0043027	0.018
475	GO:0010459	0.018
476	GO:0016278	0.018
477	GO:0016279	0.018
478	GO:0042659	0.018
479	GO:0006637	0.018
480	GO:0035383	0.018
481	GO:0048297	0.018

482	GO:0005876	0.018
483	GO:1901338	0.018
484	GO:0042054	0.018
485	GO:0036092	0.018
486	GO:0007632	0.018
487	GO:0032279	0.018
488	GO:0086011	0.018
489	GO:0032465	0.018
490	GO:0004869	0.018
491	GO:0003099	0.018
492	GO:0003321	0.018
493	GO:0010519	0.018
494	GO:0071824	0.018
495	GO:0050686	0.018
496	GO:0043028	0.018
497	GO:0086065	0.018
498	GO:0016212	0.018
499	GO:0036137	0.018
500	GO:0031720	0.018
