# Supplementary material for: The Use of Gene Ontology Term and KEGG Pathway Enrichment for Analysis of Drug Half-Life
Source: PLoS One. 2016 Oct 25;11(10):e0165496. doi: 10.1371/journal.pone.0165496 (PMC5079577; doi:10.1371/journal.pone.0165496)
Supplement: S3 Table — (PDF) [file pone.0165496.s003.pdf]

S3 Table. The MaxRel feature list with 278 KEGG pathways

Order	KEGG pathway	MI value
1	hsa04080	0.026
2	hsa00400	0.024
3	hsa05322	0.02
4	hsa04726	0.018
5	hsa00591	0.017
6	hsa05213	0.016
7	hsa00531	0.016
8	hsa04146	0.016
9	hsa00100	0.015
10	hsa00603	0.015
11	hsa04530	0.014
12	hsa04666	0.014
13	hsa00130	0.013
14	hsa04610	0.013
15	hsa00240	0.013
16	hsa04020	0.013
17	hsa04725	0.013
18	hsa00280	0.013
19	hsa03430	0.012

20	hsa05330	0.012
21	hsa00564	0.012
22	hsa03450	0.012
23	hsa04728	0.012
24	hsa05217	0.012
25	hsa00310	0.012
26	hsa05223	0.012
27	hsa05033	0.012
28	hsa04950	0.011
29	hsa04910	0.011
30	hsa05332	0.011
31	hsa00630	0.011
32	hsa04978	0.011
33	hsa00830	0.011
34	hsa05034	0.01
35	hsa00592	0.01
36	hsa03060	0.01
37	hsa00030	0.01
38	hsa03020	0.01
39	hsa00790	0.01
40	hsa04142	0.01

41	hsa00780	0.01
42	hsa05131	0.01
43	hsa05210	0.009
44	hsa04120	0.009
45	hsa03440	0.009
46	hsa05215	0.009
47	hsa00514	0.009
48	hsa04380	0.009
49	hsa05414	0.009
50	hsa05143	0.009
51	hsa04972	0.009
52	hsa00062	0.009
53	hsa04919	0.009
54	hsa05012	0.008
55	hsa00500	0.008
56	hsa04514	0.008
57	hsa00410	0.008
58	hsa00460	0.008
59	hsa00640	0.008
60	hsa04141	0.008
61	hsa00982	0.008

62	hsa04810	0.008
63	hsa04110	0.008
64	hsa00670	0.008
65	hsa04960	0.008
66	hsa00983	0.008
67	hsa04114	0.008
68	hsa05031	0.008
69	hsa04650	0.007
70	hsa05320	0.007
71	hsa04210	0.007
72	hsa04390	0.007
73	hsa00511	0.007
74	hsa05214	0.007
75	hsa00051	0.007
76	hsa04145	0.007
77	hsa00604	0.007
78	hsa05134	0.007
79	hsa04330	0.007
80	hsa03460	0.007
81	hsa05120	0.007
82	hsa00140	0.007

83	hsa05030	0.007
84	hsa05219	0.007
85	hsa05164	0.007
86	hsa05221	0.007
87	hsa00232	0.007
88	hsa04520	0.007
89	hsa05016	0.007
90	hsa00770	0.007
91	hsa04720	0.007
92	hsa05410	0.007
93	hsa03013	0.007
94	hsa02010	0.006
95	hsa04660	0.006
96	hsa04962	0.006
97	hsa05218	0.006
98	hsa00290	0.006
99	hsa04612	0.006
100	hsa05412	0.006
101	hsa00532	0.006
102	hsa05100	0.006
103	hsa05206	0.006

104	hsa00600	0.006
105	hsa00900	0.006
106	hsa00300	0.006
107	hsa00472	0.006
108	hsa00480	0.006
109	hsa04012	0.006
110	hsa04664	0.006
111	hsa05144	0.006
112	hsa04976	0.006
113	hsa04310	0.006
114	hsa04970	0.006
115	hsa03040	0.006
116	hsa00040	0.006
117	hsa05169	0.006
118	hsa00350	0.006
119	hsa05212	0.006
120	hsa03320	0.006
121	hsa00330	0.006
122	hsa04940	0.006
123	hsa05321	0.005
124	hsa04913	0.005

125	hsa04721	0.005
126	hsa04920	0.005
127	hsa00072	0.005
128	hsa05200	0.005
129	hsa04973	0.005
130	hsa04540	0.005
131	hsa05416	0.005
132	hsa04662	0.005
133	hsa00230	0.005
134	hsa00740	0.005
135	hsa05130	0.005
136	hsa05220	0.005
137	hsa00561	0.005
138	hsa00270	0.005
139	hsa05216	0.005
140	hsa04918	0.005
141	hsa05020	0.005
142	hsa04068	0.005
143	hsa03420	0.005
144	hsa04122	0.005
145	hsa05205	0.005

146	hsa00860	0.005
147	hsa04064	0.005
148	hsa04974	0.005
149	hsa04975	0.005
150	hsa04270	0.005
151	hsa00061	0.005
152	hsa05150	0.005
153	hsa04630	0.005
154	hsa00120	0.005
155	hsa04710	0.005
156	hsa04724	0.005
157	hsa00520	0.005
158	hsa03050	0.005
159	hsa04611	0.005
160	hsa04640	0.005
161	hsa00601	0.005
162	hsa00360	0.005
163	hsa05140	0.005
164	hsa00512	0.005
165	hsa05145	0.004
166	hsa05222	0.004

167	hsa04350	0.004
168	hsa05310	0.004
169	hsa00730	0.004
170	hsa04932	0.004
171	hsa05203	0.004
172	hsa04150	0.004
173	hsa04961	0.004
174	hsa04971	0.004
175	hsa03010	0.004
176	hsa04668	0.004
177	hsa04614	0.004
178	hsa05014	0.004
179	hsa00020	0.004
180	hsa00190	0.004
181	hsa04917	0.004
182	hsa05146	0.004
183	hsa04623	0.004
184	hsa05204	0.004
185	hsa04672	0.004
186	hsa04260	0.004
187	hsa05323	0.004

188	hsa05160	0.004
189	hsa04722	0.004
190	hsa04723	0.004
191	hsa04670	0.004
192	hsa05202	0.004
193	hsa00650	0.004
194	hsa00471	0.004
195	hsa04914	0.004
196	hsa04340	0.004
197	hsa04320	0.004
198	hsa04912	0.004
199	hsa04727	0.003
200	hsa00524	0.003
201	hsa05166	0.003
202	hsa03030	0.003
203	hsa00430	0.003
204	hsa04964	0.003
205	hsa00533	0.003
206	hsa05340	0.003
207	hsa04115	0.003
208	hsa00250	0.003

209	hsa04060	0.003
210	hsa04621	0.003
211	hsa00970	0.003
212	hsa04066	0.003
213	hsa00053	0.003
214	hsa04730	0.003
215	hsa04911	0.003
216	hsa04510	0.003
217	hsa00071	0.003
218	hsa00980	0.003
219	hsa03008	0.003
220	hsa00565	0.003
221	hsa00760	0.003
222	hsa05162	0.003
223	hsa04010	0.003
224	hsa04930	0.003
225	hsa05168	0.003
226	hsa00340	0.003
227	hsa00260	0.003
228	hsa03018	0.003
229	hsa00620	0.003

230	hsa03410	0.003
231	hsa00590	0.003
232	hsa05010	0.003
233	hsa00380	0.003
234	hsa05161	0.003
235	hsa04261	0.003
236	hsa00450	0.003
237	hsa03015	0.002
238	hsa00534	0.002
239	hsa04130	0.002
240	hsa05142	0.002
241	hsa01040	0.002
242	hsa00510	0.002
243	hsa05132	0.002
244	hsa04512	0.002
245	hsa04750	0.002
246	hsa04151	0.002
247	hsa00010	0.002
248	hsa00562	0.002
249	hsa04742	0.002
250	hsa05152	0.002

251	hsa00052	0.002
252	hsa04360	0.002
253	hsa05110	0.002
254	hsa04744	0.002
255	hsa04140	0.002
256	hsa04070	0.002
257	hsa04620	0.002
258	hsa04144	0.002
259	hsa04915	0.002
260	hsa05211	0.002
261	hsa04062	0.002
262	hsa04713	0.002
263	hsa04921	0.002
264	hsa04977	0.002
265	hsa04370	0.001
266	hsa04740	0.001
267	hsa04622	0.001
268	hsa00750	0.001
269	hsa00910	0.001
270	hsa00920	0.001
271	hsa00785	0.001

272	hsa05032	0.001
273	hsa05133	0.001
274	hsa04015	0.001
275	hsa03022	0.001
276	hsa00563	0.001
277	hsa04966	0
278	hsa04014	0
