# Supplementary material for: The Use of Gene Ontology Term and KEGG Pathway Enrichment for Analysis of Drug Half-Life
Source: PLoS One. 2016 Oct 25;11(10):e0165496. doi: 10.1371/journal.pone.0165496 (PMC5079577; doi:10.1371/journal.pone.0165496)
Supplement: S4 Table — (PDF) [file pone.0165496.s004.pdf]

S4 Table. Level values of the important GO terms for drugs with different half-lives

GO term ID	Compounds with half-lives less than 1 h	Compounds with half-lives between 1 and 4 h	Compounds with half-lives between 4 and 12 h	Compounds with half-lives between 12 and 24 h	Compounds with half-lives greater than 24 h
GO:0015347	1.375577	0.646757	0.346017	0.111789	0.267728
GO:0060033	0.442153	0.168464	0.044256	0.110608	0.046799
GO:0050998	0.343536	0.339371	0.579618	0.547503	0.346418
GO:0035115	0.09787	0.041094	0.108905	0.097517	0.246591
GO:0046972	0.314563	0.183653	0.064794	0.007656	0.008286
GO:0043995	0.314563	0.183653	0.064794	0.007656	0.008286
GO:0043996	0.314563	0.183653	0.064794	0.007656	0.008286
GO:0050805	0.508776	1.108798	1.696787	2.325685	1.64456
GO:0042364	0.210754	0.074688	0	0	0.050923

GO:0001533	0.169685	0.094436	0.013027	0.00677	0.013585
GO:0008504	0.301497	0.561611	1.21118	1.883765	0.987101
GO:0021853	0.067346	0.137329	0.255454	0.495538	0.348318
GO:0021830	0.067346	0.137329	0.255454	0.495538	0.348318
GO:0021894	0.067346	0.137329	0.255454	0.495538	0.348318
GO:0021534	0.18346	0.24407	0.431754	0.575736	0.351979
GO:0001965	0.146676	0.420468	0.574537	0.953871	0.535248
GO:1901386	0.102154	0.239363	0.480176	0.940826	0.516903
GO:0021924	0.185364	0.235542	0.449057	0.601267	0.353359
GO:0021930	0.185364	0.235542	0.449057	0.601267	0.353359
GO:0046341	0.202721	0.125694	0.035149	0.005042	0.009847
GO:0019992	0.209924	0.109275	0.017365	0.006118	0.011447
GO:0003881	0.35021	0.189669	0.036172	0	0.02668

GO:0090177	0.111031	0.009848	0	0.00442	0
