# Supplementary material for: The Use of Gene Ontology Term and KEGG Pathway Enrichment for Analysis of Drug Half-Life
Source: PLoS One. 2016 Oct 25;11(10):e0165496. doi: 10.1371/journal.pone.0165496 (PMC5079577; doi:10.1371/journal.pone.0165496)
Supplement: S5 Table — (PDF) [file pone.0165496.s005.pdf]

S5 Table. Level values of the important KEGG pathways for drugs with different half-lives

KEGG pathway ID	Compounds with half-lives less than 1 h	Compounds with half-lives between 1 and 4 h	Compounds with half-lives between 4 and 12 h	Compounds with half-lives between 12 and 24 h	Compounds with half-lives greater than 24 h
hsa04080	1.407994	4.377066	4.948055	7.559691	5.681731
hsa00400	0.158703	0.505508	0.19808	0.040488	0.392116
hsa05322	1.331333	0.679303	0.332357	0.527495	0.196206
hsa04726	1.237523	3.114835	3.816311	5.740711	3.77422
hsa00591	0.886691	1.723559	1.938082	2.448214	2.578431
hsa05213	1.927751	0.749995	0.811394	0.822282	1.756955
hsa00531	0.115013	0.170589	0.046895	0.059765	0.124753
hsa04146	0.522807	0.412032	0.150411	0.185949	0.222726
hsa00100	0.143563	0.119594	0.20486	0.323383	0.474581

hsa00603	0.090844	0.028328	0	0.066403	0.058018
hsa04530	0.853919	0.319677	0.299312	0.360101	0.696382
hsa04666	0.630264	0.321296	0.338858	0.293947	0.498496
hsa00130	0.151862	0.074852	0.04922	0.020316	0.140534
hsa04610	0.684774	0.963968	0.577233	0.384638	0.50288
hsa00240	0.973576	0.626382	0.434303	0.514906	0.708497
hsa04020	1.782916	2.435518	3.06363	3.724442	2.917448
hsa04725	1.139074	0.998564	1.381127	1.861255	1.574518
hsa00280	0.389166	0.26389	0.256835	0.180471	0.148599
